# Complementary and alternative therapies for tension-type headache: A protocol for systematic review and network meta-analysis

**DOI:** 10.1097/MD.0000000000025544

**Published:** 2021-04-23

**Authors:** Xixi Zhai, Sishuo Zhang, Chuancheng Li, Fei Liu, Qing Huo

**Affiliations:** a First College of Clinical Medicine, Shandong University of Traditional Chinese Medicine; b Affiliated Hospital of Shandong University of Traditional Chinese Medicine; c Zhangqiu Hospital of Traditional Chinese Medicine, Jinan, Shandong Province, China.

**Keywords:** complementary and alternative therapies, network meta-analysis, protocol, Tension-type headache

## Abstract

**Background:**

Tension-type headache (TTH) is the most common form of primary headache with high prevalence, which affects the quantity of life seriously. The pharmacological treatment of TTH is not the most effective. Meanwhile, complementary therapies and alternative therapies play an essential role in the treatment of TTH, and there is an absence of comparison between various interventions. Therefore, we propose the network meta-analysis protocol to compare the efficacy and safety of various complementary therapies and alternative therapies for TTH.

**Methods:**

From the beginning to February 2021, we will search the database to collect randomized controlled trials of complementary and alternative therapies for TTH. Two researchers will be responsible for screening retrieve documents, extracting data. The risk of bias will be assessed based on the Cochrane bias risk tool. We will use STATA16.0 and WinBUGS1.4.3 for paired meta-analysis and Bayesian network meta-analysis. The quality of evidence will be assessed using the grading of recommendations assessment development and evaluation.

**Results:**

This study will compare and rank the efficacy and safety of various complementary and alternative treatments for TTH.

**Conclusions:**

This study will provide more extensive evidence for the complementary and alternative therapies of TTH. We expect to assist clinicians and patients in choosing the optimum treatment.

**Protocol registration number:**

INPLASY202130088.

## 1 Introduction

Tension-type headache (TTH) is the most common type of primary headaches,^[1]^ with a 4:5 male-to-female ratio. In recent years, epidemiologic studies revealed that the incidence of TTH fluctuates wildly with different regions and social conditions. The lifetime prevalence rate was about 26.1% to 45%,^[2–4]^ and the 1-year prevalence rate was about 38.2% to 59.4%.^[5–8]^ The cardinal symptoms of TTH are bilateral pressing or tightening (nonpulsating) pain, no nausea, or vomiting, which is mainly seen in the occipital, temporal, or frontal part and can involve the whole head. The pain is not aggravated by daily physical activity, is often mild or moderate,^[9]^ and does not directly affect mortality.^[10]^ Thus, most of TTH is easy to be ignored, and the patients seeking medical help are less.^[11]^ Also, chronic or frequent episodic of TTH are afflicted by their persistent pain, resulting in more workdays lost and a decrease in GDP.^[12–14]^ Meanwhile, TTH can increase the risk of somnipathy, temporomandibular disorders, anxiety, depression, dementia, gastrointestinal disorders, and seriously damage human health.^[15–19]^ In conclusion, the burden of TTH exceeds that of migraine and cluster headache. Enough attention should be paid.^[20]^

The exact pathogenesis of TTH has remained relatively incomplete, and the present consensus is that peripheral mechanisms and central mechanisms are intertwined.^[21,22]^ Trigger points play a significant role in the pathogenesis of TTH. Trigger points are defined as sensitive spots where compressing or stretching can cause pain in nearby or distant parts.^[23]^ Their formation may be due to various factors, including excessive physical activity, psychological stress or joint dysfunction,^[24]^ which leads to the accumulation of bradykinin and other chemical mediators, resulting in peripheral nociceptor excitation and lower threshold,^[23]^ so that low-intensity stimulation can trigger the electrical signal of pain. Furthermore, the peripheral nociceptor threshold is affected by an inflammatory reaction and blood flow, muscle atrophy, and other factors.^[22]^ Therefore, peripheral pain mechanisms seem to be more likely to affect infrequent episodic TTH and frequent episodic TTH,^[25]^ whereas central sensitization is predominant in chronic TTH.^[26]^ Central sensitization is caused by continuous nociceptive input of the peripheral nociceptor. Besides, genetic factors and psychological factors such as depression and anxiety also affect TTH.^[27,28]^

Present therapeutic strategies for treating TTH mainly include 2 aspects: pharmacological and nonpharmacological treatments.^[29]^

Pharmacological treatments can be divided into the abortive treatments of each acute exacerbation and prophylactic treatments. TTH is generally treated with acetaminophen or ibuprofen for abortive treatments. However, frequent or excessive use of these drugs may lead to medication-overuse headache.^[30,31]^ Prophylactic treatments should be considered in patients who suffer from chronic TTH or frequent episodic TTH, reducing headaches frequency and severity. Studies have shown that the prophylactic main pharmacotherapy is tricyclic antidepressants,^[32]^ of which amitriptyline is the first drug confirmed to be effective by several clinical controlled studies and should be the first-line choice.^[33]^ Simultaneously, mirtazapine and botulinum toxin A were shown to be effective methods in treating chronic TTH. However, there was no more evidential study to confirm it.^[34,35]^ Furthermore, Chinese herbal medicines are an effective adjuvant treatment.^[36]^

Nonpharmacological treatments mainly include psychotherapy, behavior therapy, physical therapy, acupuncture therapy, usually combined with other pharmacological treatments, and their effectiveness has been confirmed. Research has shown that electromyography biofeedback therapies and relaxation training alone or in combination can reduce headache activity by nearly 50%.^[37]^ Relaxation training and physical exercise can improve the patient's sleep, stimulate vitality, and enhance subjective well-being.^[38]^ Cognitive-behavioral therapy can effectively reduce TTH activity, and it will be more useful when combined with pharmacological treatments.^[33]^ Mindfulness therapy has a positive effect on pain relief.^[39]^ Yoga may be an effective intervention for TTH.^[40]^ Massage, manipulation, and other therapies can reduce the intensity and frequency of pain, shorten the time of pain, and improve the quality of life of patients,^[41,42]^ suggesting they may be a good alternative nonpharmacological treatment for TTH. Dry needling therapy safely reduces headache intensity and frequency and shortens the duration of pain.^[43]^ Besides, acupuncture therapy has been proved effective in the long term,^[44,45]^ and transcutaneous electrical nerve stimulation (TENS) significantly reduced the severity of headache.^[46]^

Nonpharmacological treatment is a significant component of complementary and therapeutic therapies. Complementary and therapeutic therapies include electromyography biofeedback therapy, relaxation training, cognitive behavioral therapy, physical exercise, yoga, massage, management, TENS, dry needling therapy, acupuncture, Chinese herbal medicine, and so forth, which serve an essential role in the treatment of TTH.^[14]^ Therefore, how to select the best and safest intervention measures is of great significance to the treatment. However, there is no comprehensive study comparing and ranking various interventions. The network meta-analysis (NMA) can combine direct comparison with indirect comparison, which is superior to a meta-analysis comparing multiple interventions. In conclusion, the NMA will evaluate the complementary and alternative therapy of TTH and hope to provide extensive evidence for clinical practice.

## 2 Methods

The NMA will strictly follow the Preferred Reporting Items for Systematic Review and Meta-Analysis Protocols (PRISMA-P) guidelines.^[47]^

### 2.1 Study registration

The NMA has been registered on the International Platform of Registered Systematic Review and Meta-analysis Protocols (INPLASY), registration number: INPLASY202130088 (URL = https://inplasy.com/inplasy-2021-3-0088/).

### 2.2 Inclusion criteria

#### 2.2.1 Research design

The NMA will contain randomized controlled trials of complementary therapies and alternative therapies for TTH. Case reports, expert opinions, animal experiments, and review papers will not be included. Moreover, the study will only search for Chinese and English document.

#### 2.2.2 Participants

1. Patients diagnosed as TTH based on The International Classification of Headache Disorders established by the International Headache Soci ety.^[21,48,49]^
2. Patients with migraine, cluster headache, or other types of headache will be excluded.
3. No restrictions on age, race, sex, nationality, disease severity, disease duration.

#### 2.2.3 Interventions

The control group received no treatment, placebo, regular western medicine, or other conventional treatment. The treatment group received complementary and alternative therapies (electromyography biofeedback therapy, relaxation training, cognitive behavioral therapy, physical exercise, yoga, massage, management, TENS, Dry needling therapy, acupuncture, Chinese herbal medicine, and so on), and these interventions can be used alone or in combination.

#### 2.2.4 Outcomes main results

1. Primary outcomes: pain intensity (assessed by a validated tool, such as the visual analogue scale), attack frequency (number of headache attacks per evaluation interval), duration of pain (in hours).
2. Secondary outcomes: quality of life, adverse reactions.

### 2.3 Databases and search strategy

The database we will search is as follows: Cochrane Central Register of Controlled Trials, Web of Science, EMBASE, Cochrane Library, PubMed, Chinese Biomedical Literature Database (SinoMed), VIP database, China National Knowledge Infrastructure (CNKI), Wanfang Database. Also, we will track the research in the meta-analysis of TTH's complementary and alternative therapies and search for qualified studies on the World Health Organization International Clinical Trial Registry Platform (WHO ICTRP). The search period is from the establishment of the database to February 2021. Meanwhile, the search strategy will take the form of medical subject headings (MeSH) and free-text terms. The search strategy for PubMed is presented in Table 1.

**Table 1:** Search strategy for PubMed.

No.	Search terms
#1	“Tension-type headache” [MeSH Terms]
#2	Tension∗ headache[Title/Abstract] OR headache Tension∗[Title/Abstract] OR Tension∗ headaches[Title/Abstract] OR headaches Tension∗[Title/Abstract] OR Idiopathic Headache∗[Title/Abstract] OR Headache∗ Idiopathic[Title/Abstract] OR Stress Headache∗[Title/Abstract] OR Headache∗ Stress[Title/Abstract] OR Psychogenic Headache∗[Title/Abstract] OR Headache∗Psychogenic [Title/Abstract]
#3	#1 OR #2
#4	“Complementary Therapies”[MeSH Terms]
#5	Therap∗, Complementary[Title/Abstract] OR Complementary Medicine[Title/Abstract] OR Medicine Complementary[Title/Abstract] OR Alternative,Medicine[Title/Abstract] OR Medicine,Alternative[Title/Abstract] OR Therap∗,Alternative[Title/Abstract] OR Alternative,Therap∗,[Title/Abstract]
#6	#4 OR #5
#7	“Medicine, Chinese Traditional”[MeSH Terms]
#8	Medicine,Chinese Traditional[Title/Abstract] OR Traditional Chinese Medicine[Title/Abstract] OR Traditional Medicine,Chinese[Title/Abstract] OR Zhong Yi Xue[Title/Abstract] OR Chinese Traditional Medicine[Title/Abstract] OR Chinese Medicine,Traditional[Title/Abstract] OR Traditional Tongue Diagnos∗[Title/Abstract] OR Tongue Diagnos∗ Traditional[Title/Abstract] OR Traditional Tongue Assessment∗[Title/Abstract] OR Tongue Assessment,Traditional[Title/Abstract]
#9	#6 OR #7 OR #8
#10	“Drugs, Chinese Herbal”[MeSH Terms]
#11	Drugs, Chinese Herbal[Title/Abstract] OR Chinese Drugs,Plant[Title/Abstract] OR Chinese Herbal Drugs[Title/Abstract] OR Herbal Drugs,Chinese[Title/Abstract] OR Plant Extracts,Chinese[Title/Abstract] OR Chinese Plant Extracts[Title/Abstract] OR Extracts,Chinese Plant[Title/Abstract]
#12	#9 OR #10 OR #11
#13	“Physical Therapy Modalities”[MeSH Terms]
#14	Physical Therap∗[Title/Abstract] OR Physiotherap∗[Title/Abstract] OR Therapy, Physical[Title/Abstract] OR Neurophysiotherapy
#15	#12 OR #13 OR #14
#16	“Behavior Therapy”[MeSH Terms]
#17	Behavior Therap∗[Title/Abstract] OR Behavior Treatment[Title/Abstract] OR Behavior Modification∗[Title/Abstract] OR Therapy, Conditioning[Title/Abstract] OR Therapy, Behavior[Title/Abstract] OR Conditioning Therap∗[Title/Abstract] OR Treatment, Behavior[Title/Abstract] OR Modification, Behavior
#18	#15 OR #16 OR #17
#19	Acupuncture[Title/Abstract] OR Pharmacoacupuncture[Title/Abstract] OR Acupotom∗[Title/Abstract] OR Electroacupuncture[Title/Abstract] OR Moxibustion[Title/Abstract] OR Dry Needling[Title/Abstract] OR Biofeedback∗[Title/Abstract] OR Myofeedback∗[Title/Abstract] OR Feedback∗[Title/Abstract] OR Meditation[Title/Abstract] OR Yoga [Title/Abstract] OR Manipulat∗[Title/Abstract] OR Bodywork∗[Title/Abstract] OR Rolfing[Title/Abstract] OR Massage[Title/Abstract] OR Manual Therap∗[Title/Abstract] OR Therap∗, Manual[Title/Abstract] OR Faith[Title/Abstract] OR Prayer[Title/Abstract] OR exercise[Title/Abstract] OR Electric Stimulation [Title/Abstract] OR Relaxation Therapy[Title/Abstract] OR Cognitive Behavioral Therap∗
#20	#18 OR #19 OR #20
#21	“Randomized Controlled Trials as Topic”[MeSH Terms]
#22	“Randomized Controlled Trial”[Publication Type] OR Clinical Trials,Randomized[Title/Abstract] OR Trials,Randomized Clinical[Title/Abstract] OR Controlled Clinical Trials,Randomized[Title/Abstract]
#23	#21 OR #22
#24	#3 AND #20 AND #23

### 2.4 Study collection

Two researchers will independently retrieve documents according to a predetermined search strategy. We will exclude the unrelated literature by reading the abstract and title. Based on the previous step, we will further search for qualified documents by reading the full text. Then, the documents will leading-in Endnote X9 software for management. Disagreements will be solved by seeking a third independent researcher or discussing within a group.

### 2.5 Data extraction and management

Two researchers will extract data from qualified literature and record them using Microsoft Excel 2019 software. If the required data are fragmented, we will contact the corresponding author. If there is no reply, we will record this and extract the data available for analysis. We plan to extract the data as follows:

#### 2.5.1 Data basic Information

Information of literature,including title, publication date, author, journal, region, registration number of trial registration agency.

#### 2.5.2 Participants

Information of participants, including age, sex, race, source ,diagnostic criteria, inclusion criteria, exclusion criteria, disease duration, disease severity.

#### 2.5.3 Interventions

Detailed interventions information, including treatment method, frequency, dose, duration of treatment.

#### 2.5.4 Outcomes

Information of outcomes, including primary outcomes, secondary outcomes, adverse reactions.

### 2.6 Quality assessment

Two researchers will use Cochrane Collaboration's bias risk assessment tool for quality assessment.^[50]^ Each side will be classified as “low risk,” “high risk,” or “unclear.” Suppose there are differences, they will be resolved by seeking a third-party researcher.

### 2.7 Heterogeneity test

We will evaluate the heterogeneity based on the *I*^2^ value. When *I*^2^ ≤ 50%, the heterogeneity is acceptable, and the fixed-effects model will be used. When *I*^2^ > 50%, it is considered that there is significant heterogeneity in the study, and we will explore the source of heterogeneity and carry out subgroup analysis or sensitivity analysis according to the actual situation. If heterogeneity still exists, we will adopt the random-effects model.

#### 2.7.1 Sensitivity analysis

We will perform a sensitivity analysis by excluding literature to determine whether the literature affects heterogeneity. If the heterogeneity changes after excluding a document, indicating that the document affects the heterogeneity, we will analyze the reasons. On the contrary, if there is no significant change in heterogeneity, the results are reliable.

#### 2.7.2 Subgroup analysis

Assuming that there is significant statistical heterogeneity, we will perform subgroup analysis according to various heterogeneity sources. For example, the patients were grouped according to their age, sex, disease duration, and disease severity.

### 2.8 Statistical analysis

#### 2.8.1 Pairwise meta-analysis

Stata16.0 was used for paired meta-analysis to compare the literature results. Continuous data will be described by standardized mean difference or mean difference, and dichotomous data will be described by the odds ratio. Besides, a 95% confidence interval will be calculated for both, and heterogeneity will be assessed based on the *I*^2^ value.

#### 2.8.2 NMA

Stata16.0 draws the network diagram, and Bayesian NMA is realized by Bayes Markov Chain Monte Carlo (MCMC) in WinBUGS1.4.3.^[51]^ When running the WinBUGS1.4.3, we will use the Brooks Gelman Rubin method and trace graph to evaluate the convergence of iteration. If the potential scale reduction factor tends to 1, it means that the convergence is better, and the conclusion is more credible. Moreover, we will use the surface under the Cumulative Ranking Curve value to rank interventions. The closer the value is to 1, the therapeutic effect is better.^[52]^ Supposing there is a closed loop in NMA, we need to use the node splitting method to evaluate the consistency.

### 2.9 Assessment of publication bias

Supposing there are >10 researches in the meta-analysis, the publication bias will adopt the funnel plot for analysis.

### 2.10 Evaluation of evidence quality

We will use the grading of recommendations assessment development and evaluation (GRADE) tool to assess the quality of evidence and it is divided into four levels: High, medium, low, and very low.^[53]^

### 2.11 Ethics and dissemination

The protocol does not evaluate personal information or affect the patient's rights and does not require moral recognition.

## 3 Discussions

TTH is the most common type of headache, and its prevalence is on the rise. It has a negative effect on education level, income, quality of life,^[54–56]^ which is not conducive to developing the social economy. At present, the pharmacological treatments of TTH are not the best and effective. Meanwhile, complementary therapies and alternative therapies play an essential role in relieving symptoms. Since meta-analysis cannot evaluate multiple interventions simultaneously, the NMA can compare and rank various interventions to evaluate different complementary and alternative therapies’ effectiveness and safety. This study conducted extensive documents searches and used Bayesian models to assess the complementary and alternative therapies of TTH, but some unavoidable limitations still exist. For example, our data are from literature rather than randomized controlled trial, and there may be some bias. Second, we only screened Chinese and English documents, which led to publication bias. Therefore, through this study, we expect to provide more evidence for the complementary therapies and alternative therapies of THH.

## Author contributions

**Conceptualization:** Xixi Zhai, Qing Huo.

**Formal analysis:** Xixi Zhai, Chuancheng Li, Qing Huo.

**Methodology:** Sishuo Zhang. Fei Liu,

**Project administration:** Sishuo Zhang, Chuancheng Li.

**Software:** Sishuo Zhang, Chuancheng Li.

**Writing – original draft:** Xixi Zhai, Fei Liu.

**Writing – review & editing:** Xixi Zhai, Qing Huo.
